# Supplementary material for: Isomers Recognition in HPLC-MS/MS Analysis of Human Plasma Samples by Using an Ion Trap Supported by a Linear Equations-Based Algorithm
Source: Int J Mol Sci. 2023 Jul 6;24(13):11155. doi: 10.3390/ijms241311155 (PMC10342714; doi:10.3390/ijms241311155)
Supplement: Supplementary file 1 [file ijms-24-11155-s001.zip › ijms-2393686-supplementary.pdf]

Isomers Recognition in HPLC-MS/MS Analysis of Human Plasma Samples by Using an Ion Trap Supported by a Linear Equations-Based Algorithm

Marco Pallecchi, Luigi Lucio, Laura Braconi, Marta Menicatti, Silvia Dei, Elisabetta Teodori and Gianluca Bartolucci *

Neurofarba Department, Section of Pharmaceutical and Nutraceutical Sciences, University of Florence, Polo Scientifico, Via U. Schiff 6, Sesto Fiorentino, 50019 Firenze, Italy; marco.pallecchi@unifi.it (M.P.); luigi.lucio@stud.unifi.it (L.L.); laura.braconi@unifi.it (L.B.); marta.menicatti@gmail.com (M.M.); silvia.dei@unifi.it (S.D.); elisabetta.teodori@unifi.it (E.T.)

*   Correspondence: gianluca.bartolucci@unifi.it

Supplementary Material

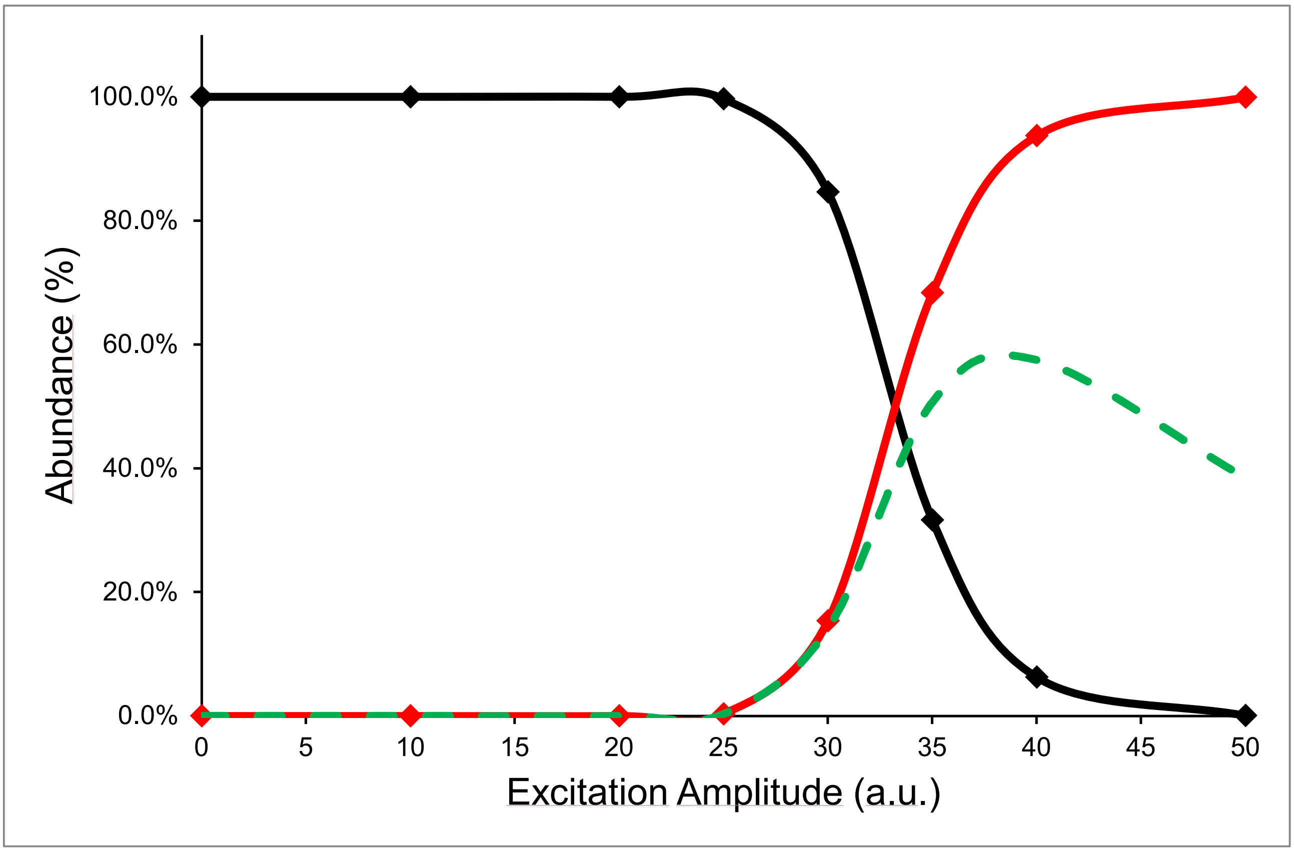

Figure S1: Precursor SY curve (black line), PiF (red line) and PiY (green dashed line) of ELF94 isomer at ExT 10 ms.

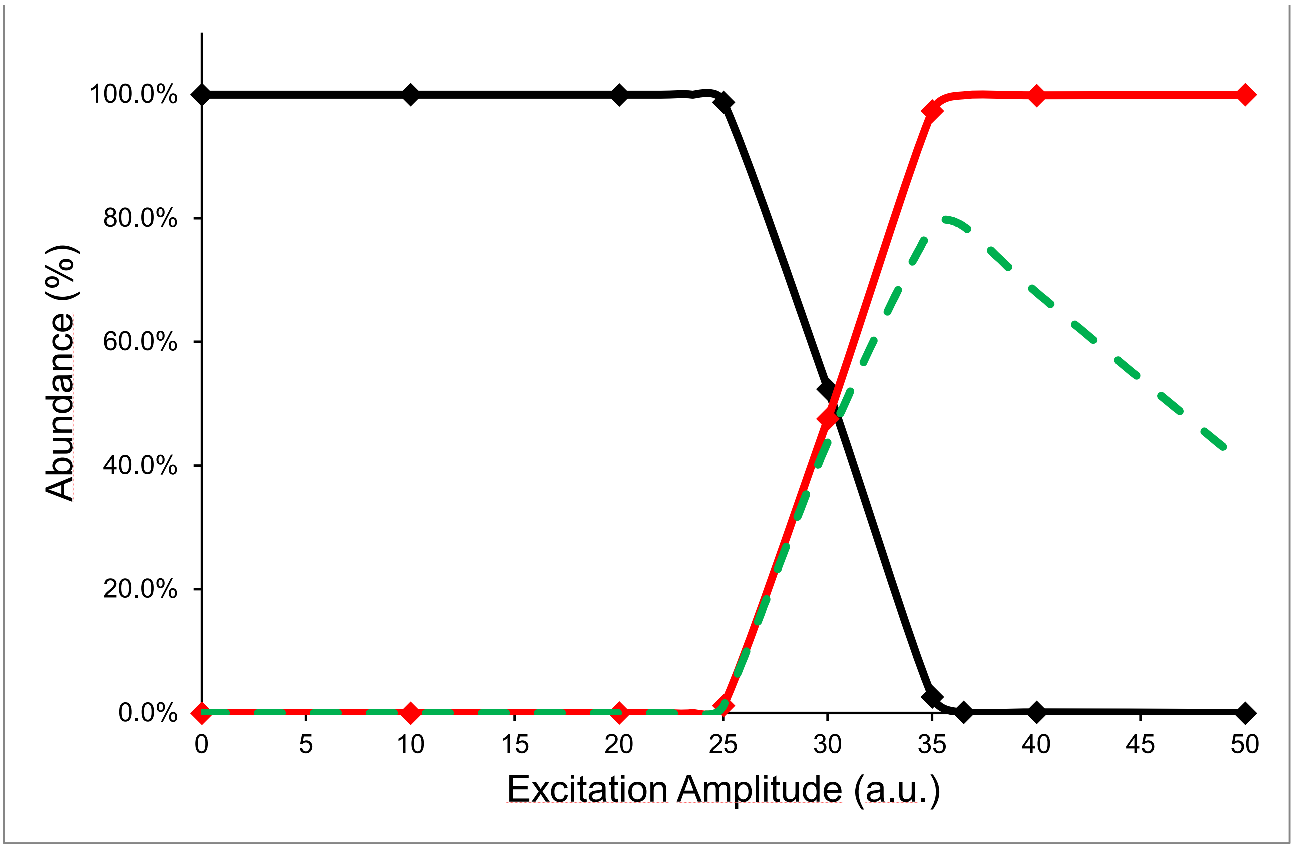

Figure S2: Precursor SY curve (black line), PiF (red line) and PiY (green dashed line) of ELF94 isomer at ExT 25 ms.

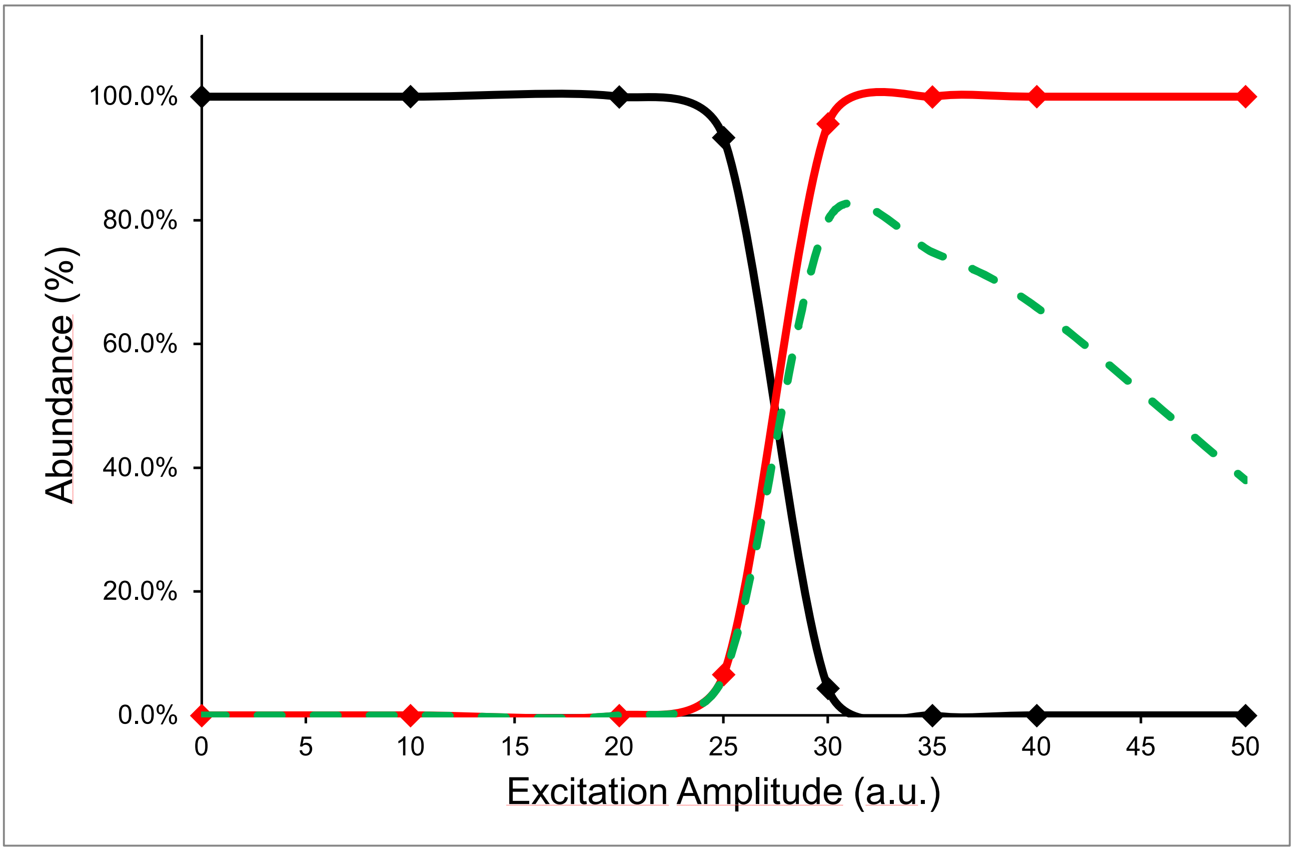

Figure S3: Precursor SY curve (black line), PiF (red line) and PiY (green dashed line) of ELF94 isomer at ExT 100 ms.

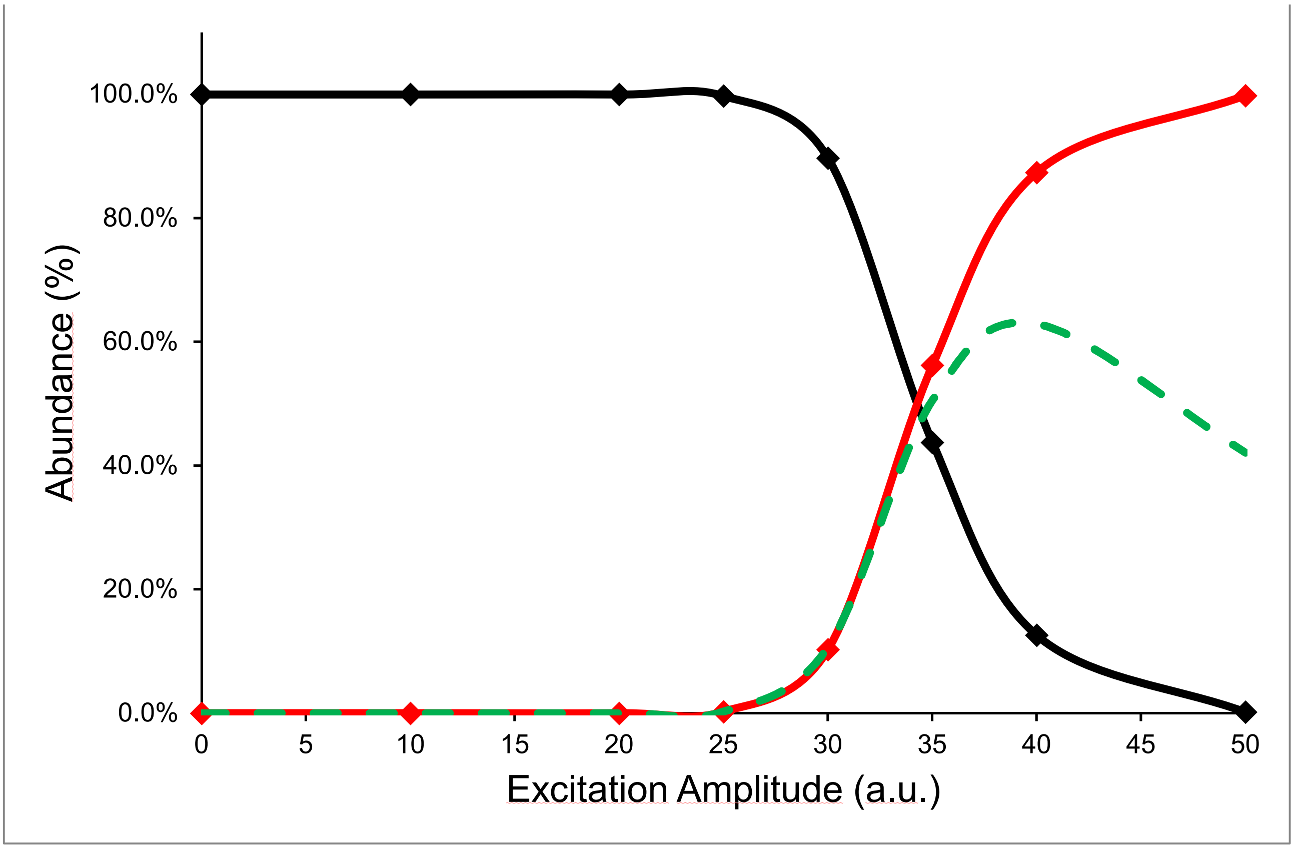

Figure S4: Precursor SY curve (black line), PiF (red line) and PiY (green dashed line) of ELF96 isomer at ExT 10 ms.

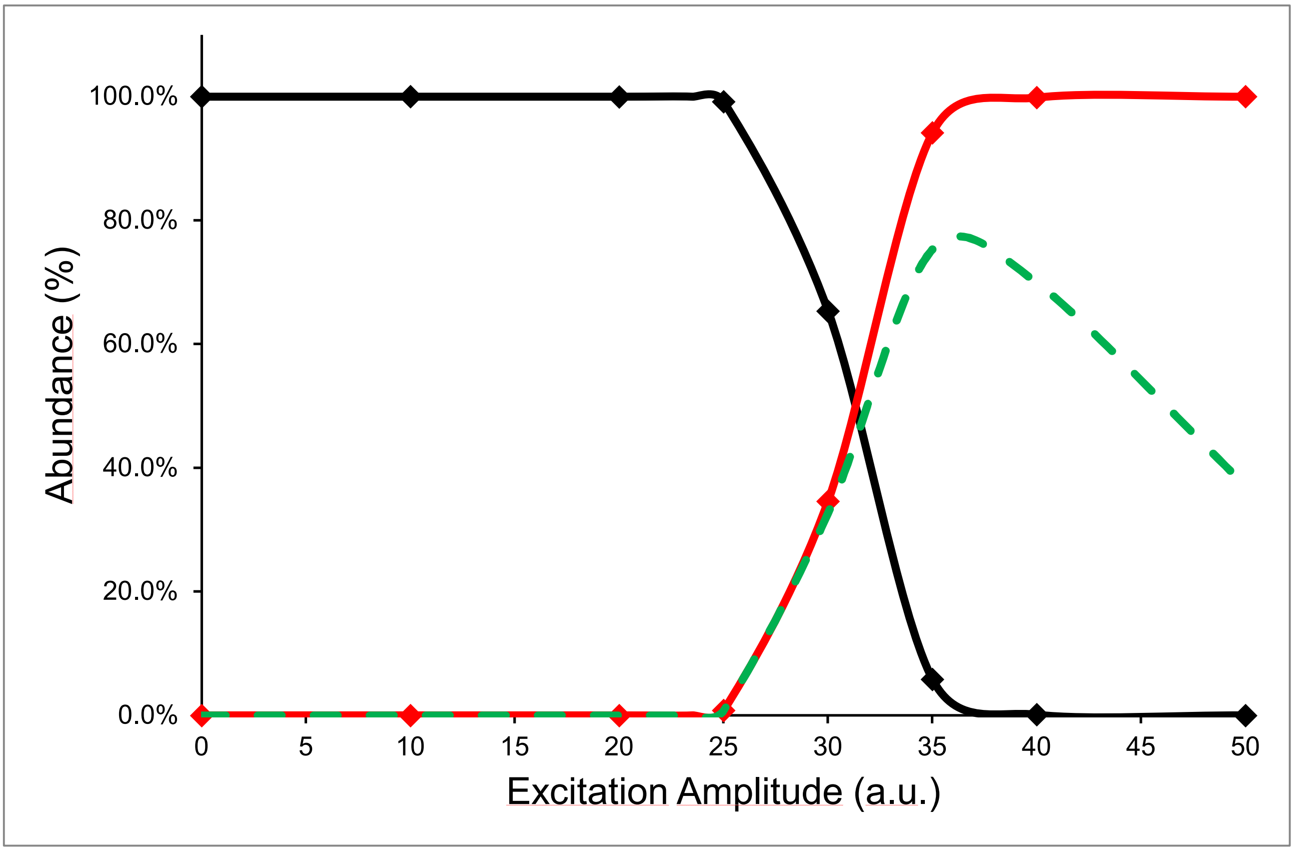

Figure S5: Precursor SY curve (black line), PiF (red line) and PiY (green dashed line) of ELF96 isomer at ExT 25 ms.

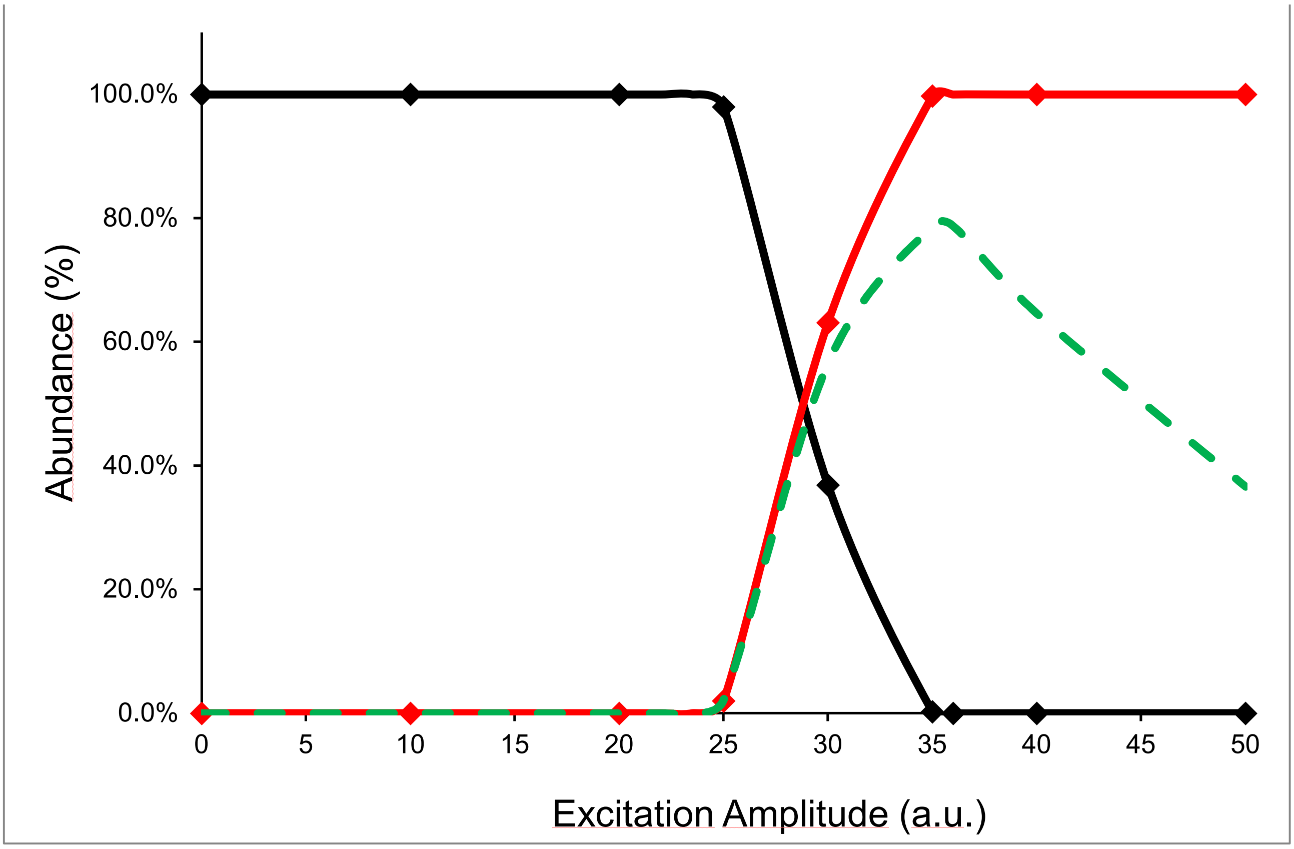

Figure S6: Precursor SY curve (black line), PiF (red line) and PiY (green dashed line) of ELF96 isomer at ExT 50 ms.

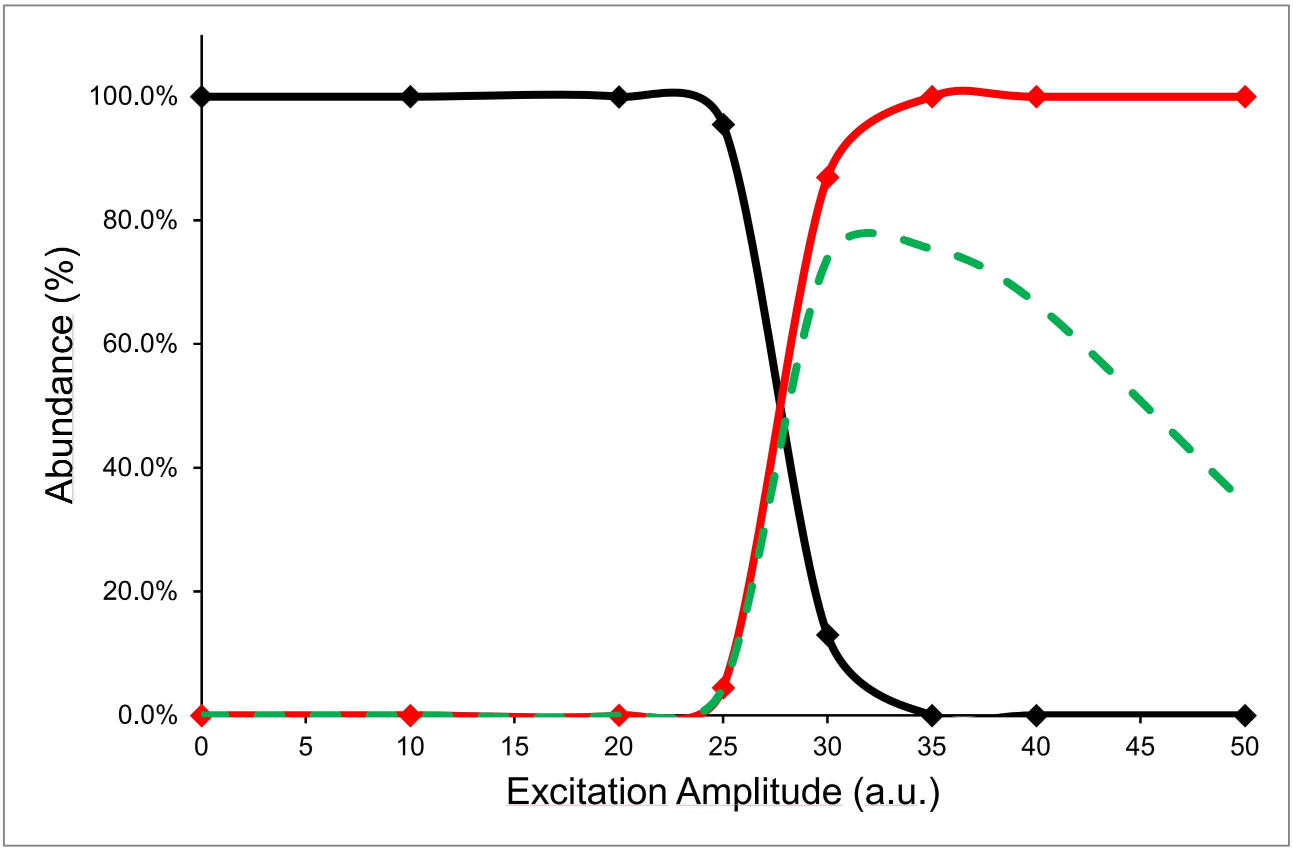

Figure S7: Precursor SY curve (black line), PiF (red line) and PiY (green dashed line) of ELF96 isomer at ExT 100 ms.

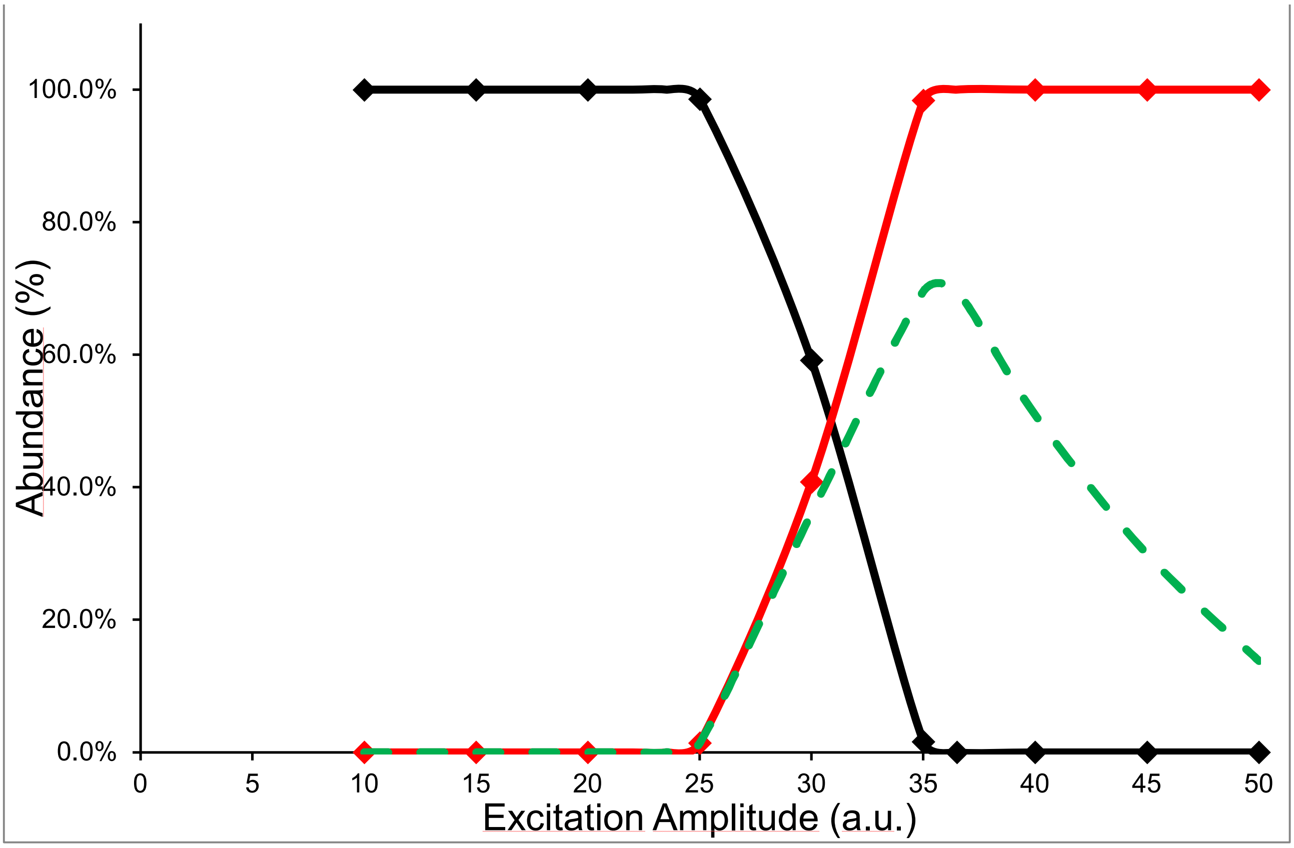

Figure S8: Precursor SY curve (black line), PiF (red line) and PiY (green dashed line) of IS at ExT 50 ms

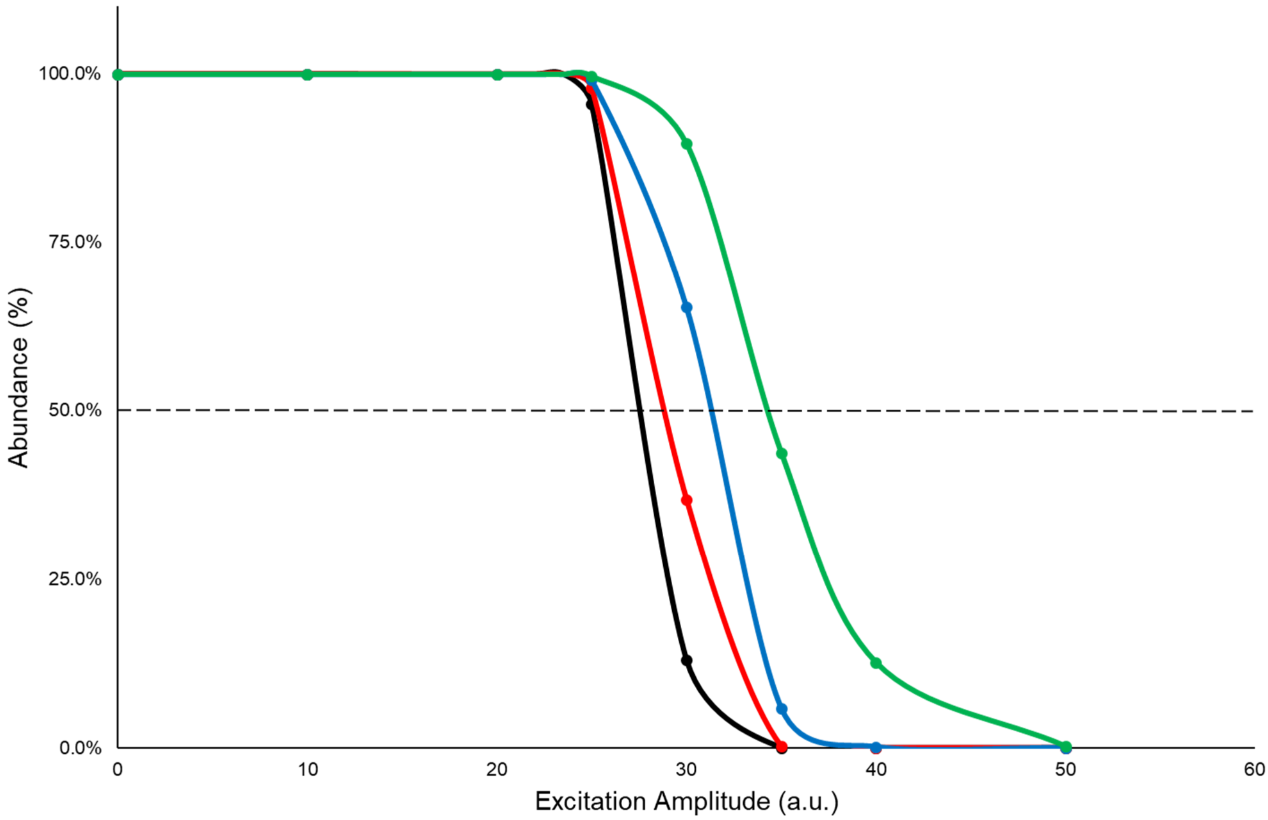

Figure S9: Comparison between precursor SY curves of ELF96 by using ExT values of 10 ms (green line), 25 ms (blue line), 50 ms (red line) and 100 ms (black line).

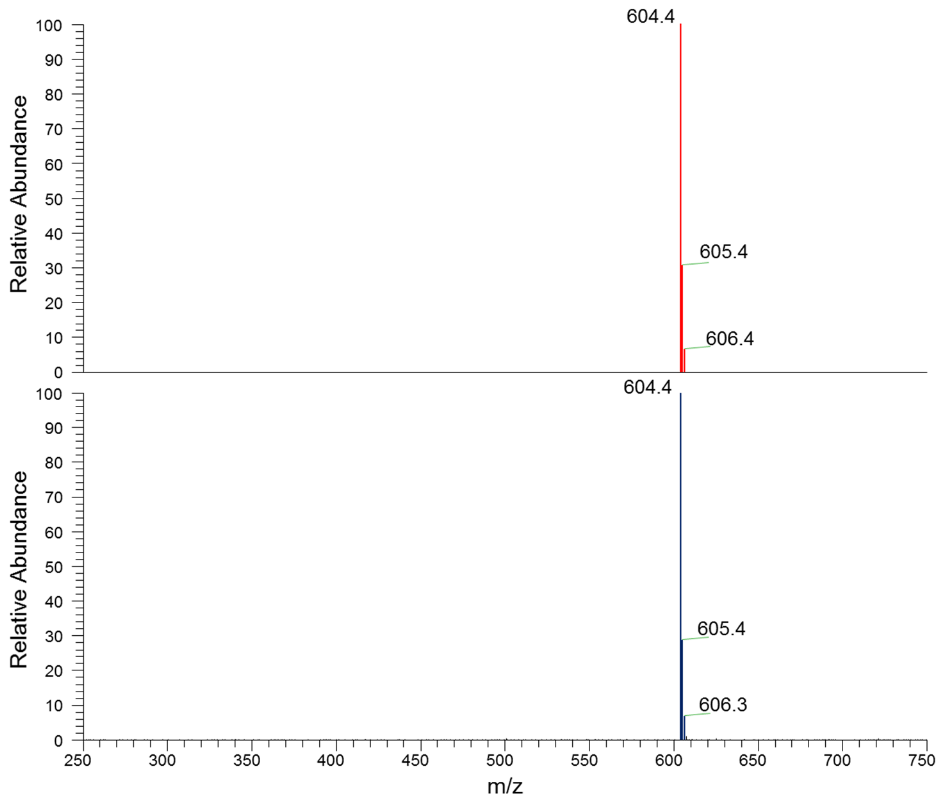

Figure S10: ESI-MS spectra of the ELF94 (red spectrum) and ELF96 (blue spectrum) isomers.

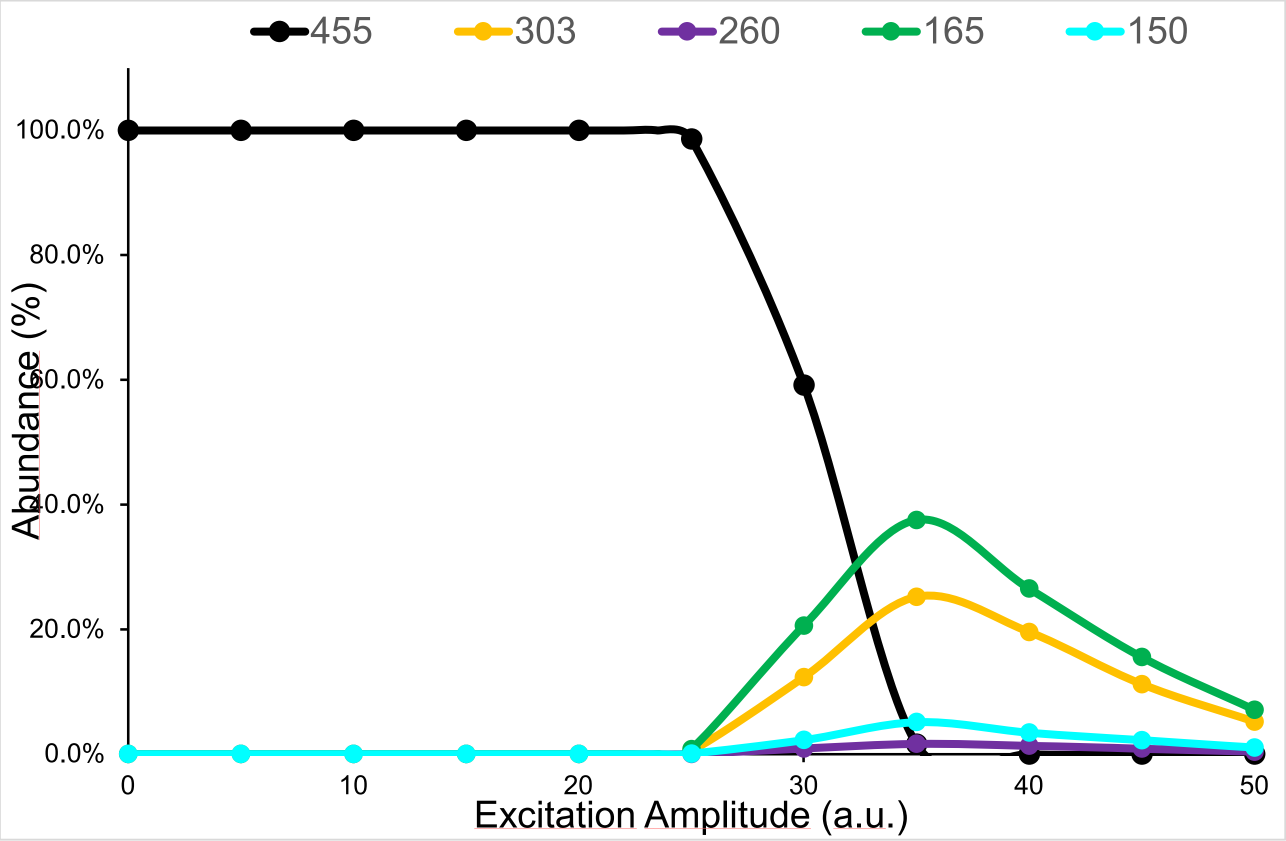

Figure S11: The breakdown curves of IS.

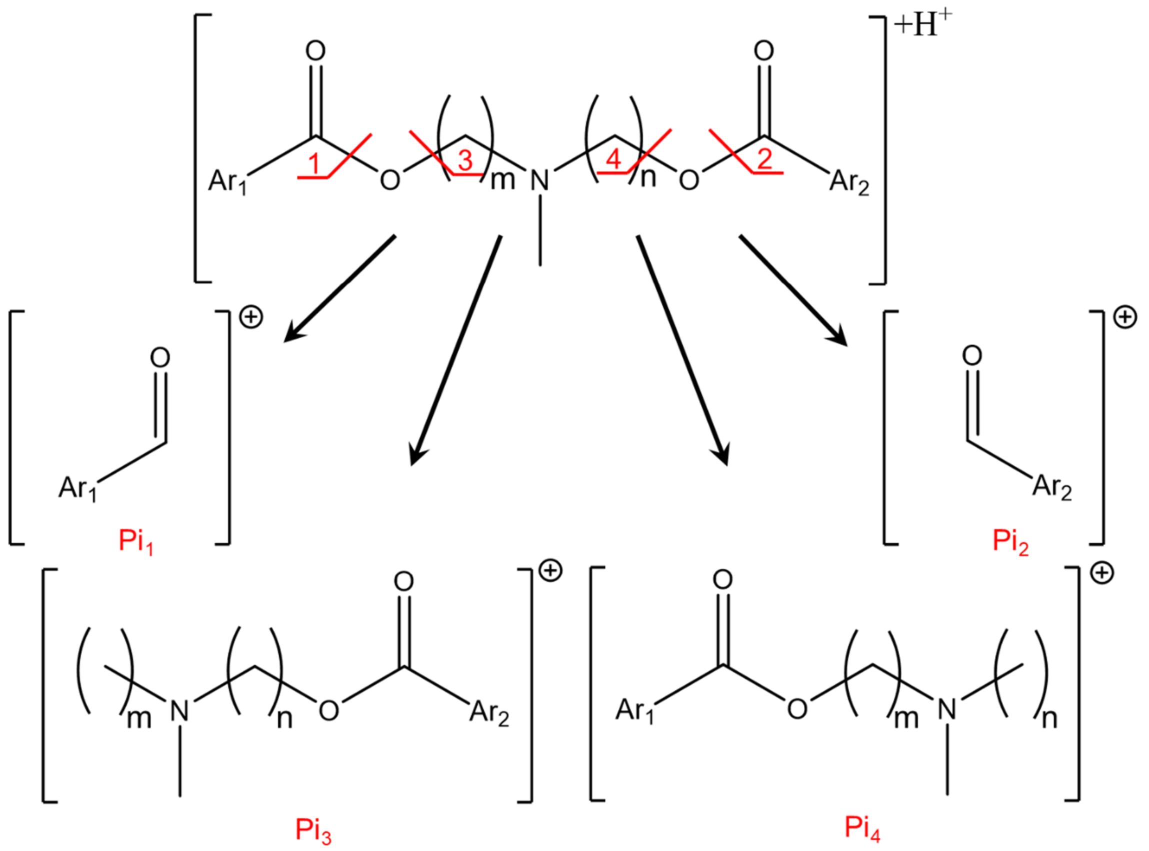

Figure S12: Common fragmentation pathways proposed for the ELF94 and ELF96 isomers.

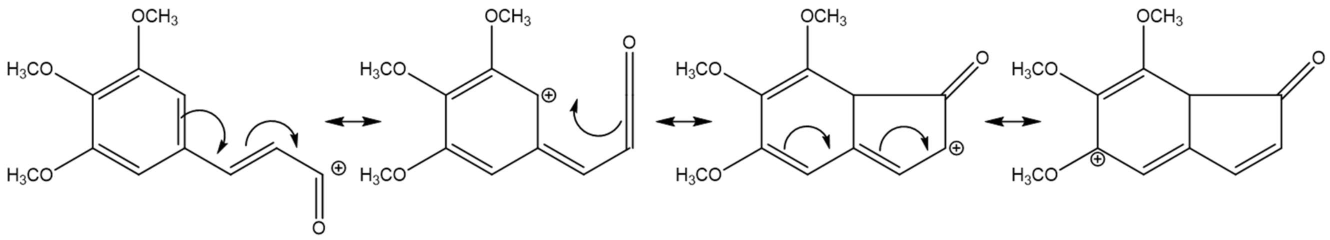

Chemical Formula: $C_{12}H_{13}O_4^+$

Exact Mass: 221.08

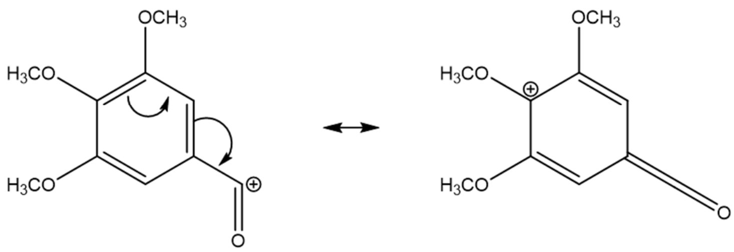

Chemical Formula: $C_{10}H_{11}O_4^+$

Exact Mass: 195.07

Figure S13: The proposed molecular structures of Pi_1 (221 *m*/*z*) and Pi_2 (195 *m*/*z*).

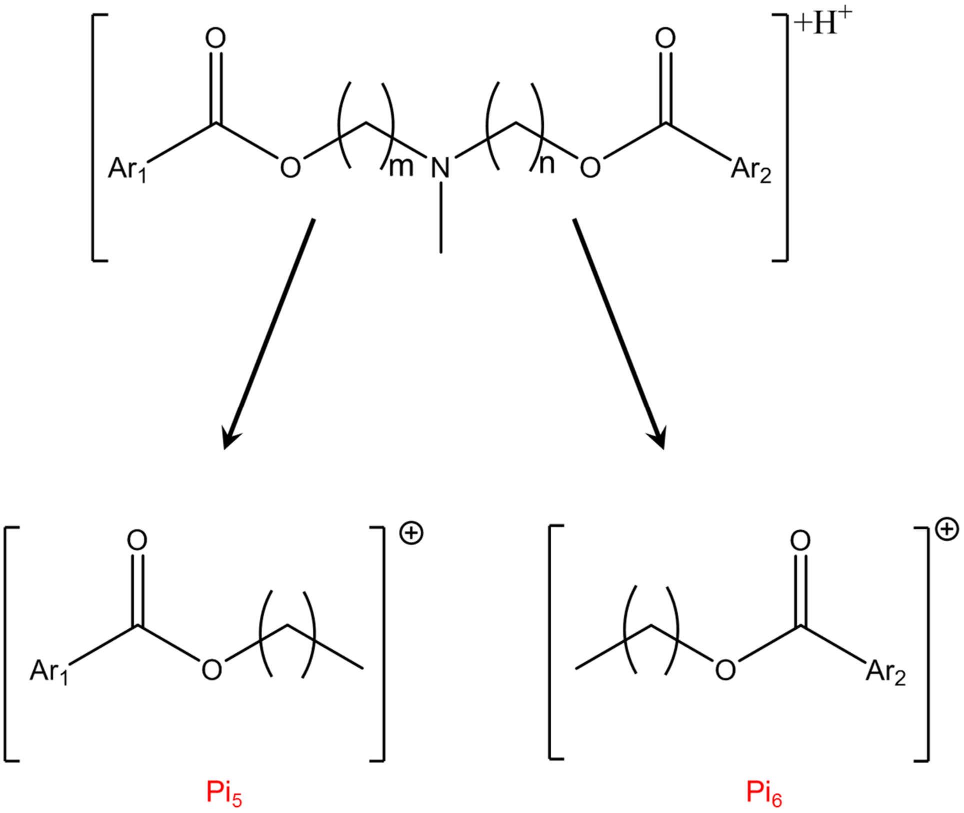

Figure S14: The fragmentation hypothesis for the formation of Pi5 and Pi6 of the ELF94 and ELF96 isomers.

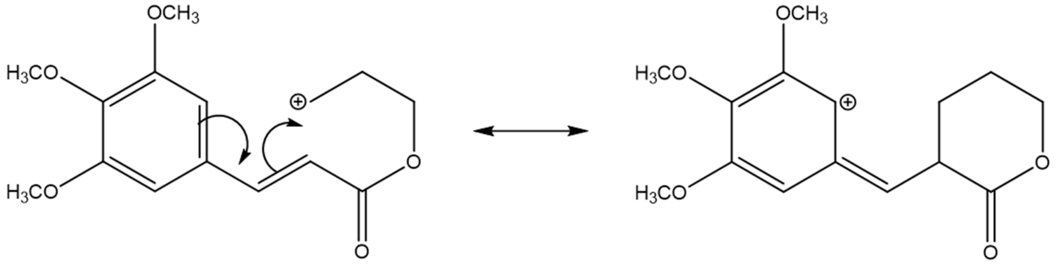

Chemical Formula: $C_{15}H_{19}O_5^+$
Exact Mass: 279.12

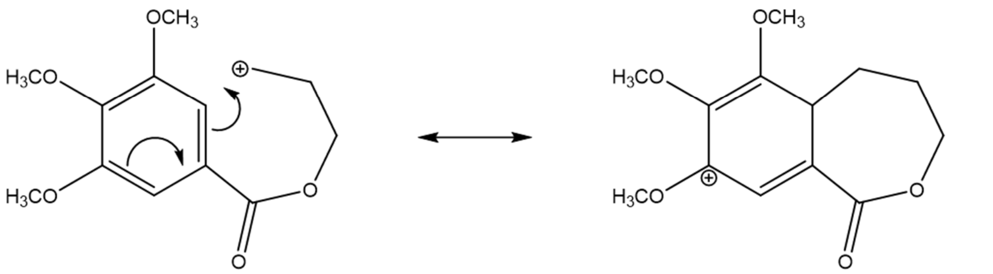

Chemical Formula: $C_{13}H_{17}O_5^+$
Exact Mass: 253.11

Figure S15: The propose molecular structures of Pi5 and Pi6.

Table S1. The chromatographic parameters retention times (Rt) ± Error (2 standard deviation or 2 SD), base peaks width (Base Width), efficiency (N) and related relative standard deviation (RSD %) for each analyte obtained with the proposed HPLC-MS/MS approach. The void time measured for the chromatographic system used was 0.51 min.

	**Rt (min.)**	**2 SD (min.)**	**Base Width (min.)**	**2 SD (min.)**	**N**	**RSD (%)**
IS	3.02	0.02	0.18	0.02	4721	9%
ELF94	3.42	0.02	0.17	0.02	5776	10%
ELF96	3.44	0.02	0.17	0.02	5844	9%

LEDA algorithm

LEDA$_{All}$ matrix for isomers speciation.

$$\begin{bmatrix} Area_{Pi_1}/Area_{Ri} = \left({}^{Pi_1}/_{Ri}\right)_{isomer\,1}[\%]_{isomer\,1} + \left({}^{Pi_1}/_{Ri}\right)_{isomer\,2}[\%]_{isomer\,2} \\ Area_{Pi_3}/Area_{Ri} = \left({}^{Pi_3}/_{Ri}\right)_{isomer\,1}[\%]_{isomer\,1} + \left({}^{Pi_3}/_{Ri}\right)_{isomer\,2}[\%]_{isomer\,2} \\ Area_{Pi_4}/Area_{Ri} = \left({}^{Pi_4}/_{Ri}\right)_{isomer\,1}[\%]_{isomer\,1} + \left({}^{Pi_4}/_{Ri}\right)_{isomer\,2}[\%]_{isomer\,2} \\ Area_{Pi_5}/Area_{Ri} = \left({}^{Pi_5}/_{Ri}\right)_{isomer\,1}[\%]_{isomer\,1} + \left({}^{Pi_5}/_{Ri}\right)_{isomer\,2}[\%]_{isomer\,2} \\ Area_{Pi_6}/Area_{Ri} = \left({}^{Pi_6}/_{Ri}\right)_{isomer\,1}[\%]_{isomer\,1} + \left({}^{Pi_6}/_{Ri}\right)_{isomer\,2}[\%]_{isomer\,2} \\ Area_{Pi_7}/Area_{Ri} = \left({}^{Pi_7}/_{Ri}\right)_{isomer\,1}[\%]_{isomer\,1} + \left({}^{Pi_7}/_{Ri}\right)_{isomer\,2}[\%]_{isomer\,2} \end{bmatrix} \quad \text{(S1)}$$

$$\begin{bmatrix} Area_{Pi_1}/Area_{Ri} \\ Area_{Pi_3}/Area_{Ri} \\ Area_{Pi_4}/Area_{Ri} \\ Area_{Pi_5}/Area_{Ri} \\ Area_{Pi_6}/Area_{Ri} \\ Area_{Pi_7}/Area_{Ri} \end{bmatrix} = A$$

$$\begin{bmatrix} \left({}^{Pi_1}/_{Ri}\right)_{isomer\,1} & \left({}^{Pi_1}/_{Ri}\right)_{isomer\,2} \\ \left({}^{Pi_3}/_{Ri}\right)_{isomer\,1} & \left({}^{Pi_3}/_{Ri}\right)_{isomer\,2} \\ \left({}^{Pi_4}/_{Ri}\right)_{isomer\,1} & \left({}^{Pi_4}/_{Ri}\right)_{isomer\,2} \\ \left({}^{Pi_5}/_{Ri}\right)_{isomer\,1} & \left({}^{Pi_5}/_{Ri}\right)_{isomer\,2} \\ \left({}^{Pi_6}/_{Ri}\right)_{isomer\,1} & \left({}^{Pi_6}/_{Ri}\right)_{isomer\,2} \\ \left({}^{Pi_7}/_{Ri}\right)_{isomer\,1} & \left({}^{Pi_7}/_{Ri}\right)_{isomer\,2} \end{bmatrix} = K$$

$$\left[[\%]_{Isomer\,1} \quad [\%]_{isomer\,2}\right] = X$$

Traspose K matrix = K'

Inverse $(K \times K')$ = $(K \times K')^{-1}$

$$X = (A \times K') \times (K \times K')^{-1} \quad \text{(S2)}$$

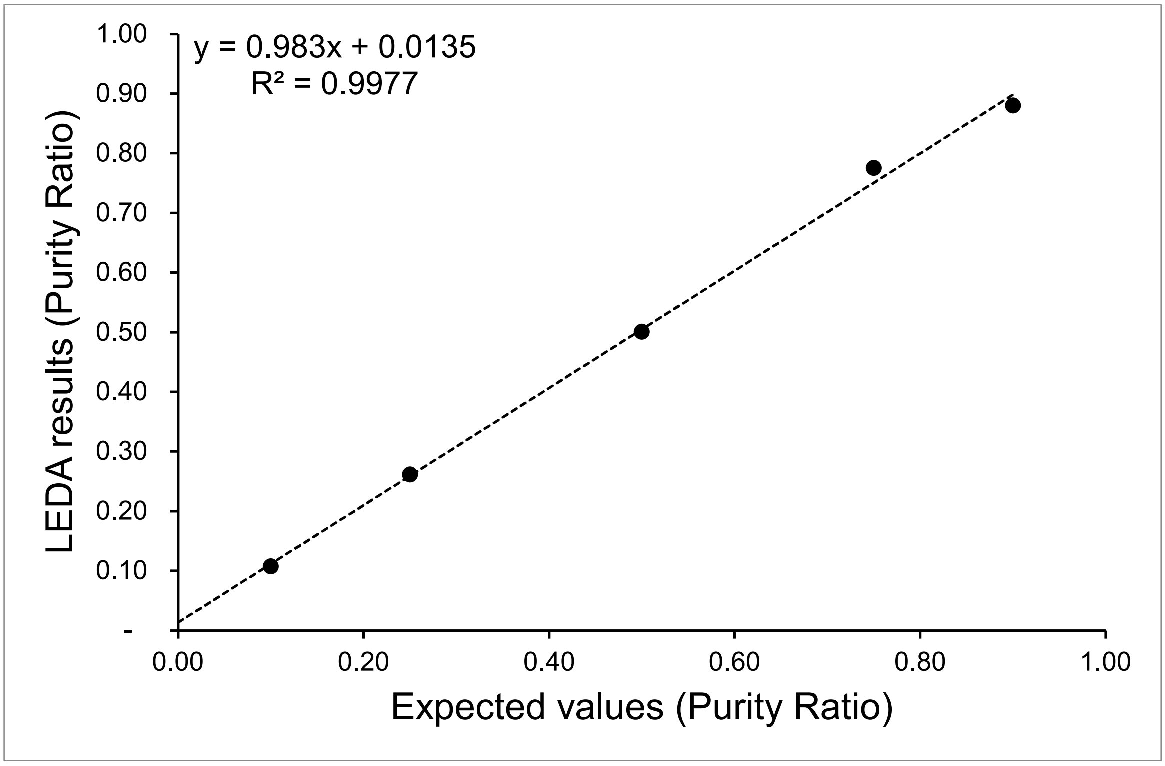

Figure S16: Validation plot obtained for the ELF94 isomer by processing the standard mixtures with $LEDA_{All}$ matrix.

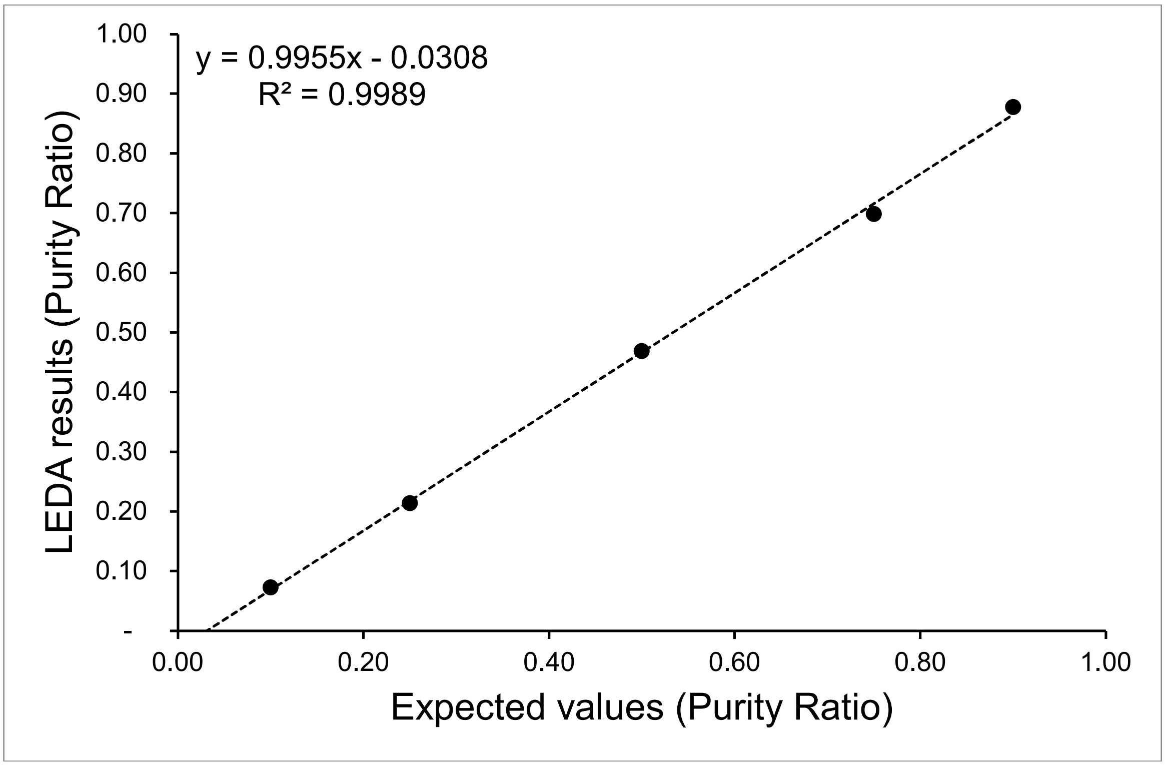

Figure S17: Validation plot obtained for the ELF96 isomer by processing the standard mixtures with $LEDA_{All}$ matrix. The t-test (95% confidence interval with n-2=3 degrees of freedom) confirmed that the calculated value of the slope (0.9955) is not statistically different from 1 and the intercept is not statistically different from 0.

The LEDA reconstructed chromatographic profiles of all the sample involved in this study were reported below

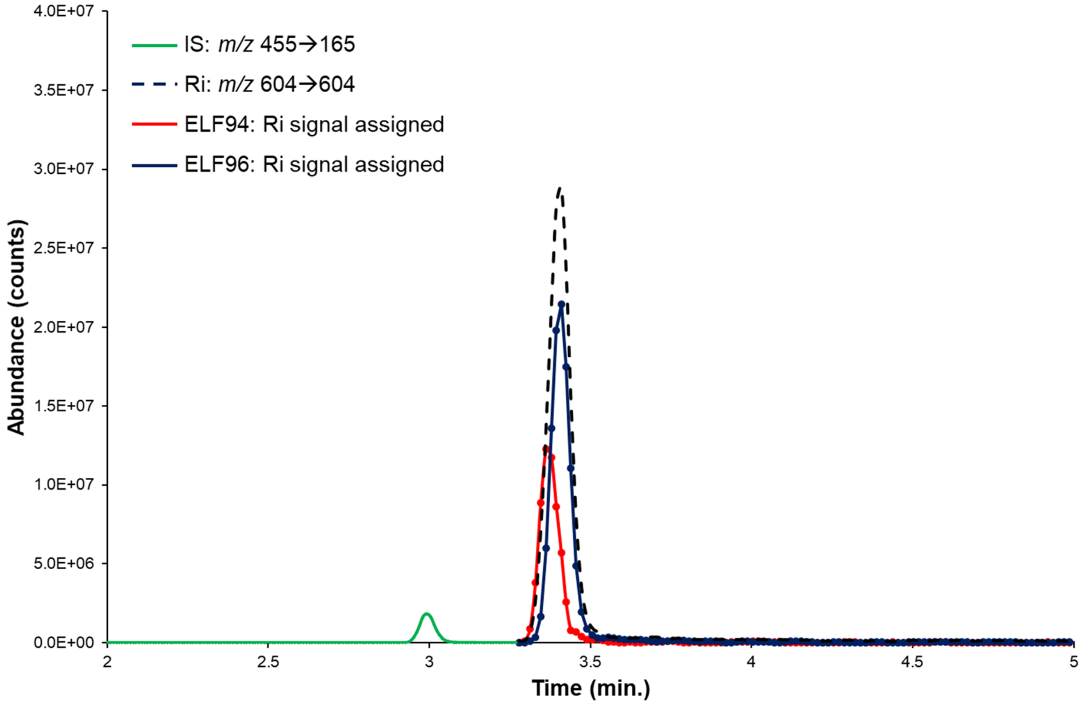

Figure S18: The LEDA reconstructed chromatographic profiles of the human plasma sample spiked with the mixture of the ELF94 (red line) and ELF96 (blue line) isomers at the incubation time 30 minutes. The IS (green line) and Ri (black dotted line) signals were also reported.

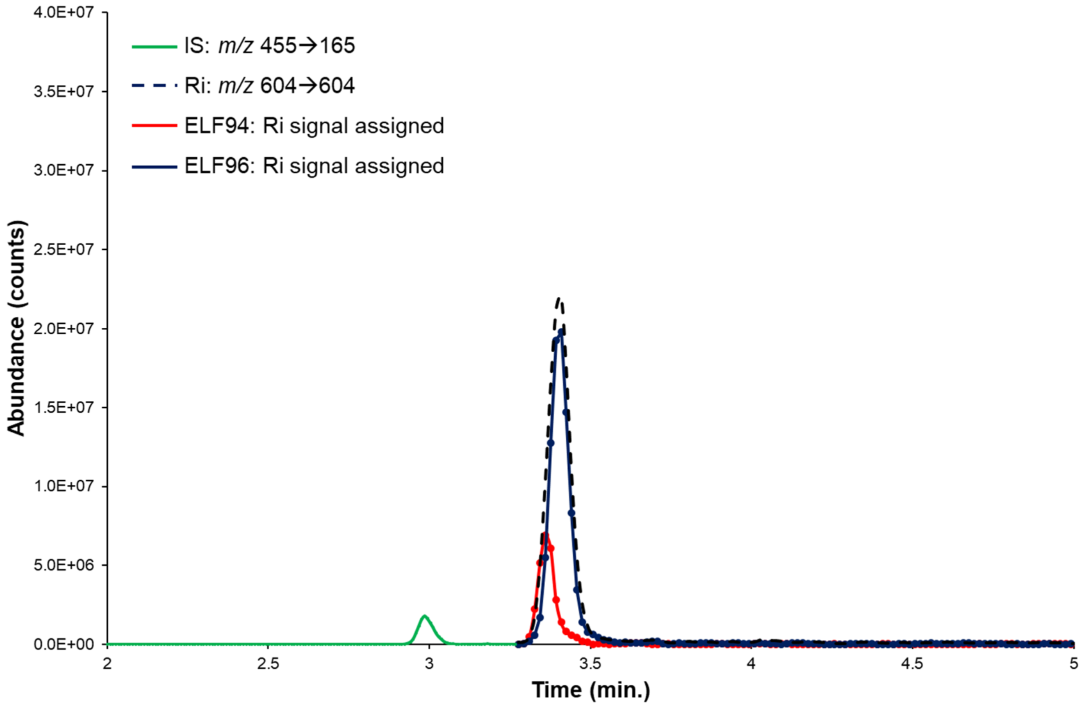

Figure S19: The LEDA reconstructed chromatographic profiles of the human plasma sample spiked with the mixture of the ELF94 (red line) and ELF96 (blue line) isomers at the incubation time 60 minutes. The IS (green line) and Ri (black dotted line) signals were also reported.

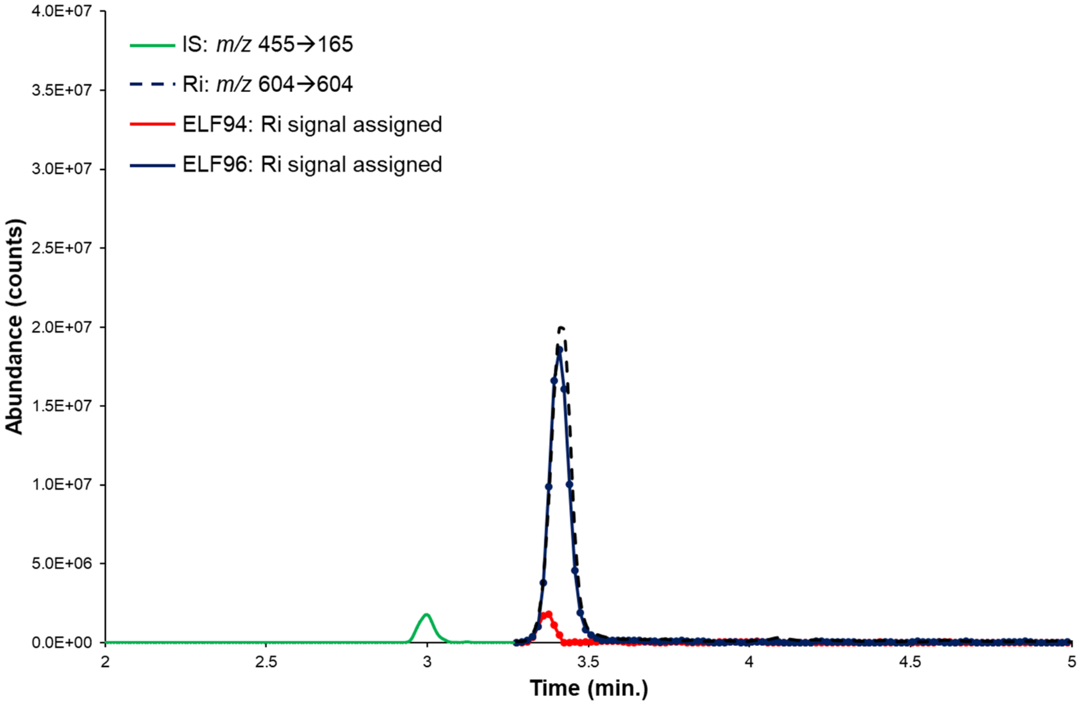

Figure S20: The LEDA reconstructed chromatographic profiles of the human plasma sample spiked with the mixture of the ELF94 (red line) and ELF96 (blue line) isomers at the incubation time 120 minutes. The IS (green line) and Ri (black dotted line) signals were also reported.

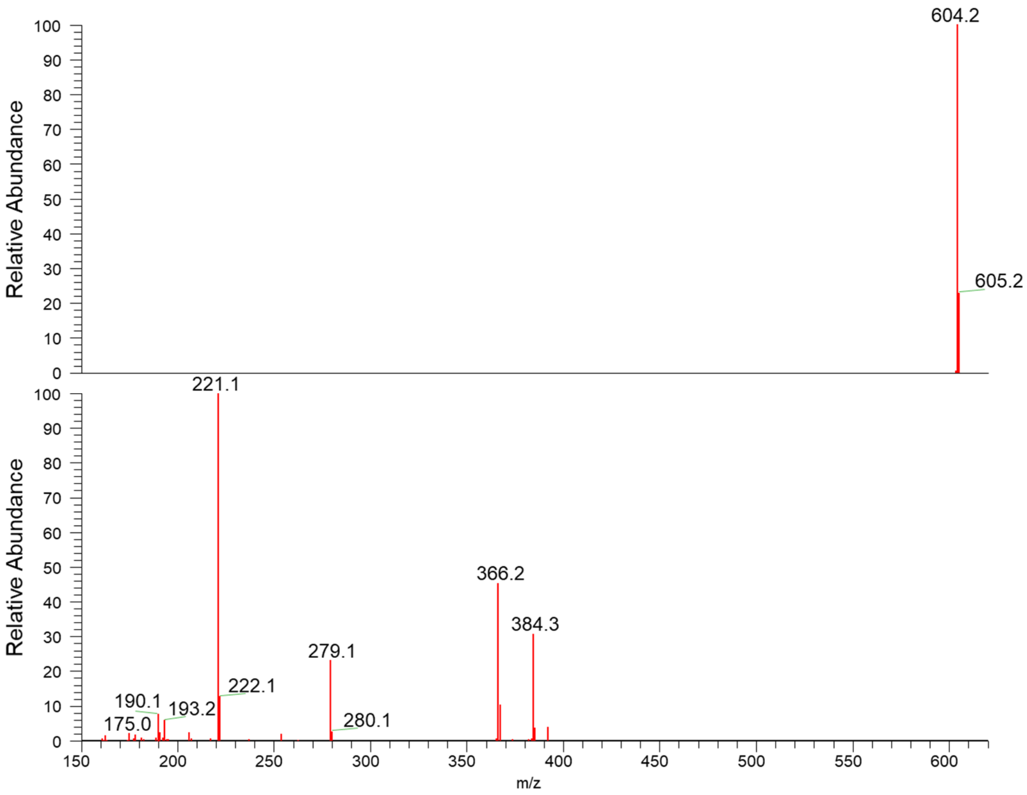

Figure S21: The spectra of MS/MS event 1 (top) and MS/MS event 2 (bottom) extracted from time segment 2 of proposed HPLC-MS/MS method by the analysis of a calibration level 100 ng ml^{-1} of ELF94.

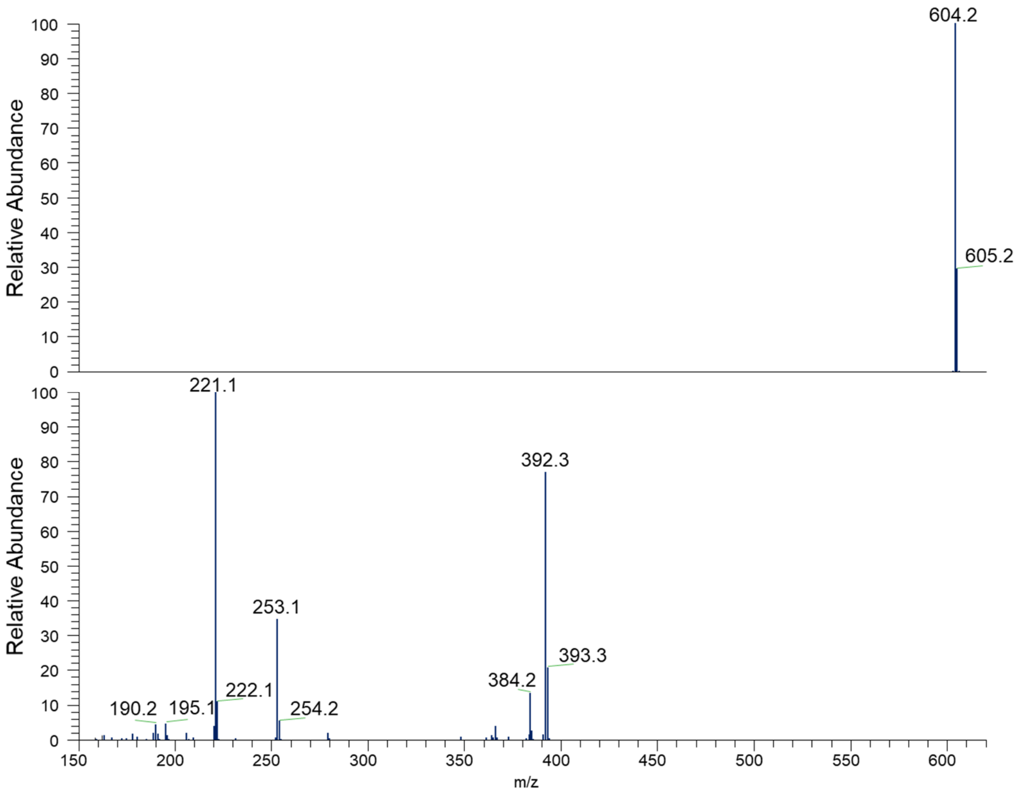

Figure S22: The spectra of MS/MS event 1 (top) and MS/MS event 2 (bottom) extracted from time segment 2 of proposed HPLC-MS/MS method by the analysis of a calibration level 100 ng ml^{-1} of ELF96.

Table S2. Time segments, MS/MS parameters and the selected of ionic signals of the IS and each isomer used for quantitative determination.

Compound	Time segment	Time segment (min.)	Precursor Ion (m/z)	MS/MS event	Quan. ion (m/z)	ExA (a.u.)
IS	1	0.00-3.27	455	IS	165	35
ELF94<br>ELF96	2	3.28-10.00	604	Ri	604	20
				Pi_1	221	35

Table S3. The results of calibration curves obtained for Ri and Pi_1 signals of each isomer, defined as linear regressions parameters (slope and y-intercept), the determination coefficient (R^2) and the estimated LOD and LOQ values.

Compound	MS/MS signal	Slope (PAR/ng mL^{-1})	y-Intercept (PAR)	R^2	LOD (ng mL^{-1})	LOQ (ng mL^{-1})
ELF94	Ri	0.256	0.243	0.999	1.8	5.4
	Pi_1	0.098	0.064	0.999	2.1	6.4
ELF96	Ri	0.265	0.069	0.999	1.0	3.2
	Pi_1	0.073	0.010	0.999	1.5	4.5

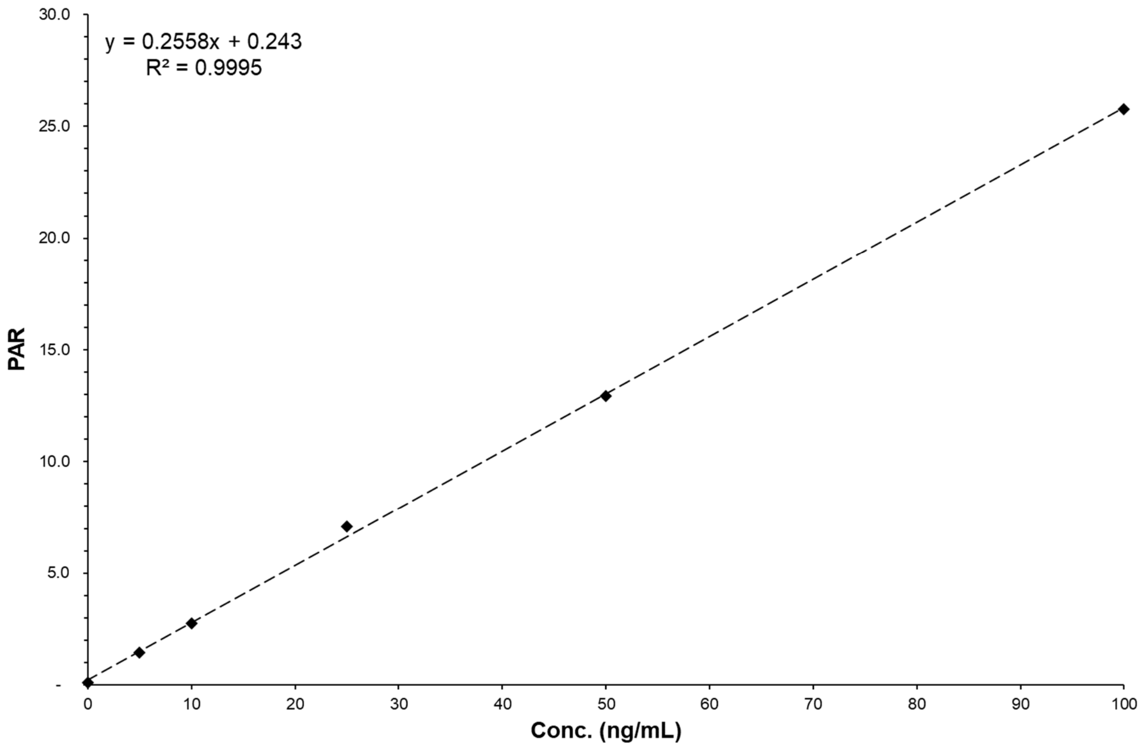

Figure S23: Internal standard **c**alibration curve considering the peak area ratio (PAR) between Ri vs IS signals obtained by analysis of calibration solutions of ELF94 isomer with HPLC-MS/MS proposed method.

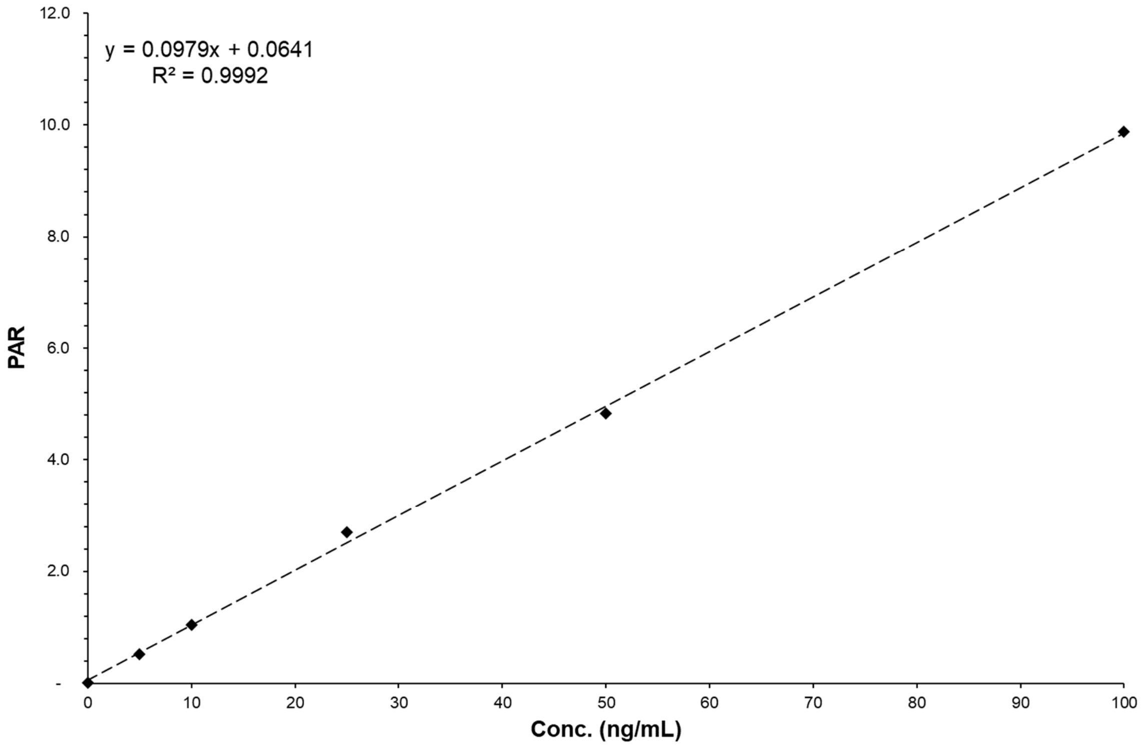

Figure S24: Internal standard **c**alibration curve considering the peak area ratio (PAR) between Pi_1 vs IS signals obtained by analysis of calibration solutions of ELF94 isomer with HPLC-MS/MS proposed method.

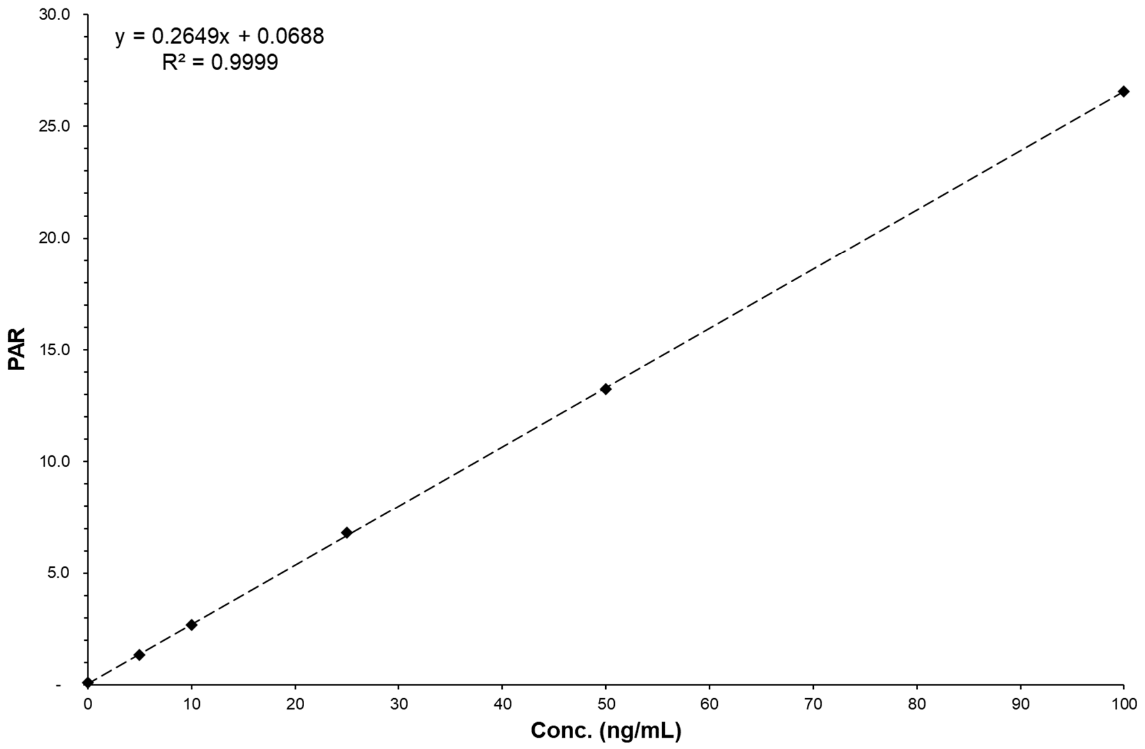

Figure S25: Internal standard **c**alibration curve considering the peak area ratio (PAR) between Ri vs IS signals obtained by analysis of calibration solutions of ELF96 isomer with HPLC-MS/MS proposed method.

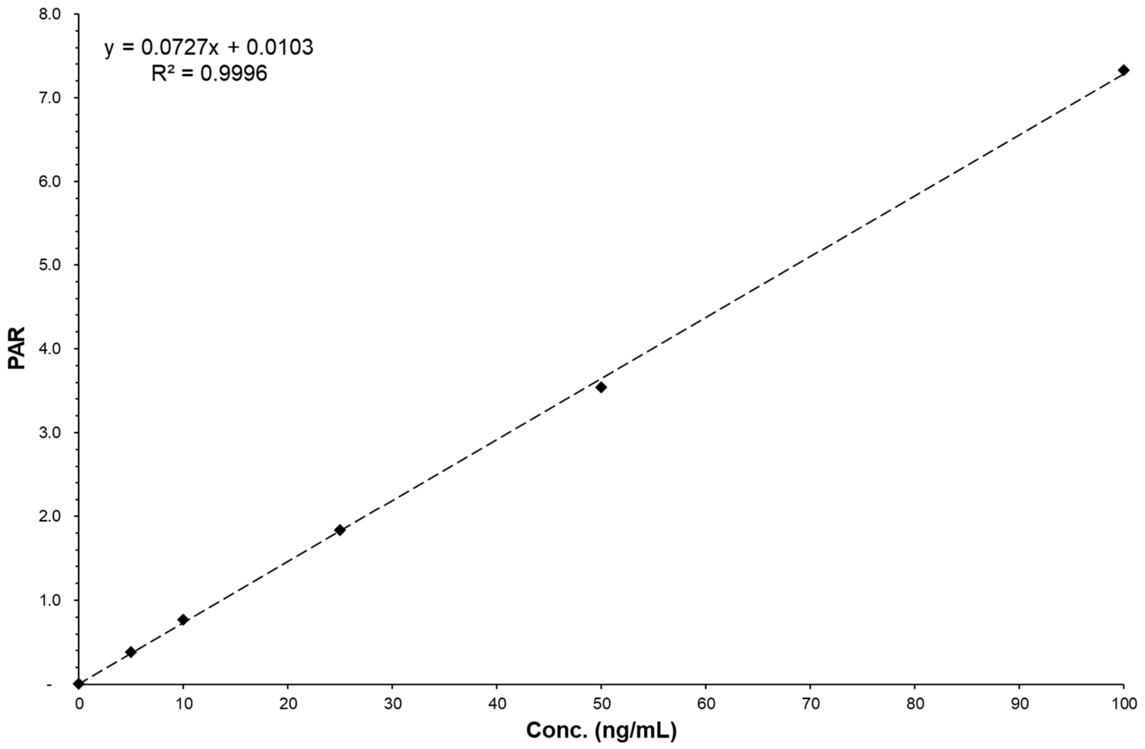

Figure S26: Internal standard **c**alibration curve considering the peak area ratio (PAR) between Pi_1 vs IS signals obtained by analysis of calibration solutions of ELF96 isomer with HPLC-MS/MS proposed method.

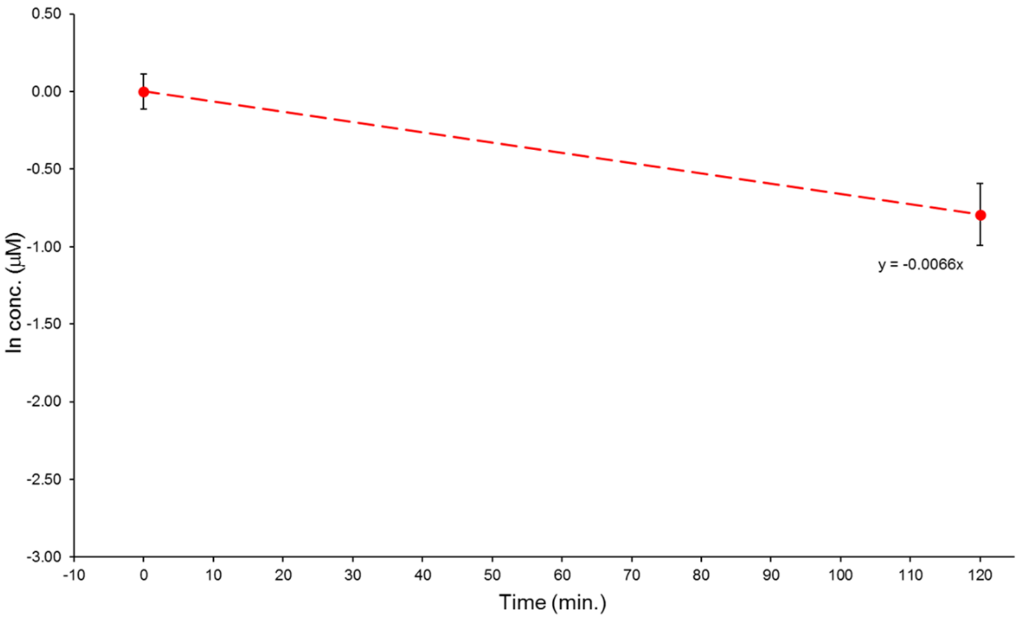

Figure S27: Degradation plots obtained by analysis, with proper HPLC-MS/MS method, of the human plasma samples spiked with the KEE reference compound.
